# A Reliable Method Based on Liquid Chromatography–Tandem Mass Spectrometry for the Simultaneous Quantification of Neurotransmitters in *Caenorhabditis elegans*

**DOI:** 10.3390/molecules28145373

**Published:** 2023-07-13

**Authors:** Ann-Kathrin Weishaupt, Laura Kubens, Lysann Ruecker, Tanja Schwerdtle, Michael Aschner, Julia Bornhorst

**Affiliations:** 1 Food Chemistry, Faculty of Mathematics and Natural Sciences, University of Wuppertal, Gaußstraße 20, 42119 Wuppertal, Germany; weishaupt@uni-wuppertal.de (A.-K.W.); laura.kubens@googlemail.com (L.K.); lysann-ruecker@gmx.net (L.R.); 2 TraceAge—DFG Research Unit on Interactions of Essential Trace Elements in Healthy and Diseased Elderly (FOR 2558), Berlin-Potsdam-Jena-Wuppertal, 14558 Nuthetal, Germany; tanja.schwerdtle@bfr.bund.de; 3 Inorganic Chemistry, Faculty of Mathematics and Natural Sciences, University of Wuppertal, Gaußstraße 20, 42119 Wuppertal, Germany; 4 German Federal Institute for Risk Assessment (BfR), Max-Dohrn-Straße 8-10, 10589 Berlin, Germany; 5 Department of Molecular Pharmacology, Albert Einstein College of Medicine, Bronx, NY 10032, USA; michael.aschner@einsteinmed.org

**Keywords:** mass spectrometry, liquid chromatography, neurotransmitters, neurodegenerative diseases, *C. elegans*

## Abstract

Neurotransmitters like dopamine (DA), serotonin (SRT), γ-aminobutyric acid (GABA) and acetylcholine (ACh) are messenger molecules that play a pivotal role in transmitting excitation between neurons across chemical synapses, thus enabling complex processes in the central nervous system (CNS). Balance in neurotransmitter homeostasis is essential, and altered neurotransmitter levels are associated with various neurological disorders, e.g., loss of dopaminergic neurons (Parkinson’s disease) or altered ACh synthesis (Alzheimer’s disease). Therefore, it is crucial to possess adequate tools to assess precise neurotransmitter levels, and to apply targeted therapies. An established in vivo model to study neurotoxicity is the model organism *Caenorhabditis elegans* (*C. elegans*), as its neurons have been well characterized and functionally are analogous to mammals. We have developed a liquid chromatography–tandem mass spectrometry (LC-MS/MS) method including a sample preparation assuring neurotransmitter stability, which allows a simultaneous neurotransmitter quantification of DA, SRT, GABA and ACh in *C. elegans*, but can easily be applied to other matrices. LC-MS/MS combined with isotope-labeled standards is the tool of choice, due to its otherwise unattainable sensitivity and specificity. Using *C. elegans* together with our analytically validated and verified method provides a powerful tool to evaluate mechanisms of neurotoxicity, and furthermore to identify possible therapeutic approaches.

## 1. Introduction

Neurotransmitters are messenger molecules transmitting excitation between neurons across chemical synapses, which enable the brain to sense perceptions and coordinate complex behavior [1]. Here, the most important neurotransmitters, dopamine (DA), serotonin (SRT), γ-aminobutyric acid (GABA) and acetylcholine (ACh) will be discussed, as their dysregulation, among others, is associated with several neurological diseases. DA regulates body movement control, as well as memory function and cognition [2,3]. The most common DA-associated neurodegenerative disorder is Parkinson’s disease (PD), which is associated with the progressive loss of dopaminergic neurons in the substantia nigra [4] and is characterized, among other things, by the presence of alpha-synuclein inclusions (Lewy Bodies) [5]. SRT acts as a neurohormone controlling the function of several peripheral organs and modulates mood, cognition, sleep, learning and anxiety [6,7]. Hypofunction of serotonergic neurons is associated with depression, and disturbances in SRT levels lead to anxiety disorders [7]. GABA, among other functions, regulates blood pressure and heart rate. In addition, it binds to receptors at inhibitory synapses, thus decreasing neuronal excitability [8]. The balance between excitation and inhibition is a requisite for proper neural function; as a consequence, a disequilibrium contributes to neurodegeneration [9]. The cholinergic system, including in particular the neurotransmitter ACh, is known to be required for a variety of critical physiological activities, such as attention, learning and memory [10]. A decreased activity of choline acetyltransferase (ChAT) and the subsequent altered ACh synthesis are correlated with an increased formation of ß-amyloid (Aβ) plaques in the brains of patients with Alzheimer’s disease (AD) [11]. Furthermore, a deficiency of ChAT, choline uptake and ACh secretion are concomitant symptoms of neuronal loss associated with learning deficits and memory loss [12]. Therefore, the analysis of basal levels of neurotransmitters is an essential tool for neurotoxicity assessment, especially in terms of neurodegenerative diseases such as PD and AD. In addition, neurotransmitter ratios are of great interest, as they interact and depend on each other, and in most neurodegenerative diseases the entire neurotransmitter system is disturbed [13,14]. In brief, it is crucial to have the ability to determine which neurotransmitter(s) are impaired, in order to apply targeted therapies.

Neurotransmitter quantification in mouse tissue, such as the brain [15] or cerebrospinal fluid [16], can be employed to assess the neurodegenerative potential of chemical or physical agents that may be harmful, as well as to identify therapeutic strategies. However, animal experiments provoke great ethical debate, requiring novel model organisms to substitute and complement animal experiments for testing neurodegenerative potentials. For this purpose, zebrafish (*Danio rerio*), flies (*Drosophila melanogaster*) and worms are commonly used [17,18]. The nematode *Caenorhabditis elegans* (*C. elegans*) constitutes a distinguished in vivo model featuring a well-elucidated nervous system. All neurons are well characterized and mapped over the worm body, and they are structurally and functionally similar to mammals [19]. Furthermore, in *C. elegans,* orthologs are present for 60–80% of human genes related to various diseases, including neurodegenerative disorders [20]. Therefore, *C. elegans* is a well-established model organism in the field of neurotoxicity and neurodegeneration. In addition, worms are easily genetically manipulated, providing a variety of mutants, especially for PD [21,22] and AD [23,24].

Neurotoxicity in *C. elegans* is predominantly assessed by behavioral assays. Commonly performed assays include that of the basal slowing response, which examines dopamine-dependent behavior in the presence of food [25], the determination of serotonin-dependent pharyngeal pumping [26], the synaptic transmission at neuromuscular junctions using the aldicarb-induced paralysis assay [27] and the assessment of functional changes in locomotion [25]. Additionally, genetically modified worms with fluorescence tags in neurons have been used to study neurodegeneration via fluorescence microscopy [28,29]. However, these techniques have the limitation, among others, of not being able to quantify absolute neurotransmitter levels. Furthermore, the majority of assays, such as that of basal slowing, are focused on a solitary neurotransmitter, in this case, DA. Other assays, namely those of locomotion, are mediated by several neurotransmitters, such as acetylcholine and dopamine; these provide broader outcomes, but can be problematic in interpretation. So far, only Schumacher et al. have assessed DA and SRT [30], but, to date, GABA and ACh have not been quantified in *C. elegans*. Therefore, a method is required for the simultaneous quantification of multiple neurotransmitters in *C. elegans*.

The demands of such a technique are challenging, as the analysis must be specific for the individual neurotransmitters and, on the other hand, requires good sensitivity, as the basal levels of neurotransmitters are low. In addition, neurotransmitters display poor stability. Methods do already exist to quantify neurotransmitters in a variety of matrices by electrochemical detection [31,32], fluorescence detection [33,34] or fluorescent dyes [35,36,37]. However, here, we opted for liquid chromatography–tandem mass spectrometry (LC-MS/MS) as the preferred choice due to its high sensitivity and unmatched specificity, and given its propensity to detect distinctive mass transitions of each analyte, and therefore, its capacity for unequivocal identification. In addition, mass spectrometry allows the use of isotope-labeled standards, which correspond analogously to their respective analyte throughout the entire analytical procedure, from sample preparation to detection. The combination of mass spectrometry and isotope-labeled standards of target analytes is a top-notch technique for the analysis of several biological samples [38]. In recent years, a handful of LC-MS/MS-based methods have been published to quantify neurotransmitters. These refer almost exclusively to mouse [15,16] and rat [39,40] brain tissue and mostly do not provide sufficient LOQs for neurotransmitter quantification in model organisms like *C. elegans*. Only Tufi et al. present an LC-MS/MS analysis in zebrafish *Danio rerio* [41], while Barata et al. published a method for neurotransmitter and related metabolites quantification in *Daphnia magna* [42]. A tool for the simultaneous quantification of neurotransmitters, especially GABA and ACh, in *C. elegans* with sufficient sensitivity has yet to be reported.

Here, we aim to present an established and validated LC-MS/MS-based method, which allows the simultaneous quantification of neurotransmitters, specifically DA, SRT, GABA and ACh, in *C. elegans*. A new extraction protocol assured stability and high recovery for all four analytes. The use of isotope-labeled standards and LC-MS/MS analysis in multiple-reaction-monitoring mode provided an unequivocal identification, as well as specificity of all analytes and greater sensitivity compared to other techniques. As method validation parameters, the linear range, limit of detection (LOD), limit of quantification (LOQ), accuracy, recovery and precision were assessed. Further, we analyzed neurotransmitter profiles of transgenic *C. elegans* strains with altered neurotransmitter homeostasis and characterized their synaptic transmission by the aldicarb-induced paralysis assay in order to corroborate the analytical LC-MS/MS data.

## 2. Results

### 2.1. Method Development for Neurotransmitter Quantification via LC-MS/MS

**Figure 1:**
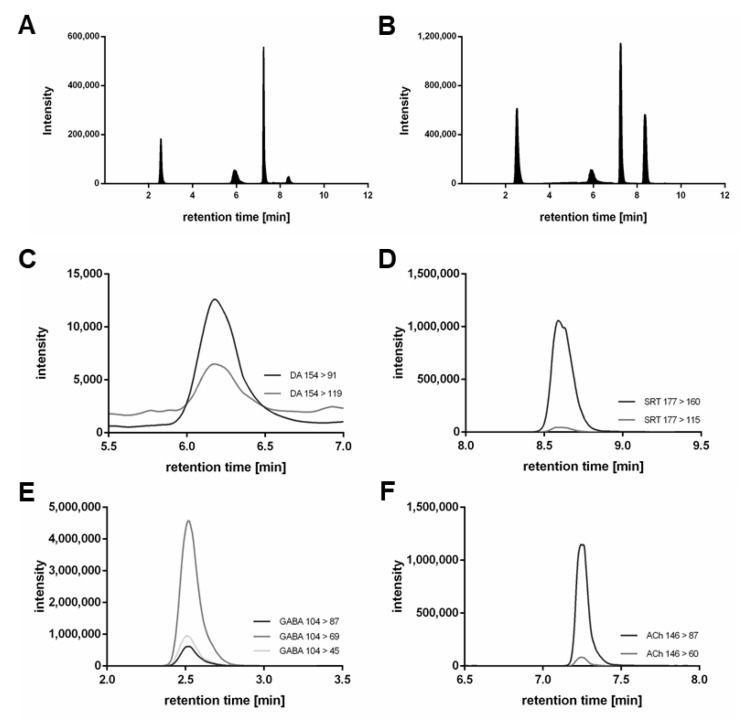
sMRM chromatograms of all analytes (**A**) and their respective deuterated internal standards (**B**) (25 nM of DA_d4_, 25 mMSRT_d4_, 500 nM of GABA_d6_ and 25 nM ACh_d4_) in *C. elegans* worm homogenate (wildtype). (**A**,**B**) only the quantifier mass transitions (Table 1) of DA, SRT, GABA, ACh and the accordant internal standards are presented. The most intensive mass transitions (listed in Table 1) of DA (*m*/*z* 154 > 137 not found in matrix) (**C**), SRT (**D**), GABA (**E**) and ACh (**F**) are displayed in matrix.

The aim of the chromatography was to establish a baseline-separated elution for all analytes, as well as maximum sensitivity with subsequent mass spectrometric detection. Different solvents (MeOH and ACN) were tested, with ACN demonstrating sharper peaks, lower noise and quicker elution of all analytes when we used the YMC-Triart PFP column. ACN modified with 10 mM FA resulted in a higher response compared to 5 mM FA. For further optimization, the column temperature was varied (20–40 °C), with 30 °C leading to the best result. A gradient of a total of 12 min (including equilibration) was generated with the following retention times for all analytes and their respective deuterated internal standards (used for internal calibration and unambiguous identification): GABA—2.50 min, DA—5.92 min, ACh—7.22 min and SRT—8.38 min. The respective chromatograms of the quantifiers of all analytes and all internal standards in a *C. elegans* matrix (wildtype) are shown in Figure 1.

**Figure 2:**
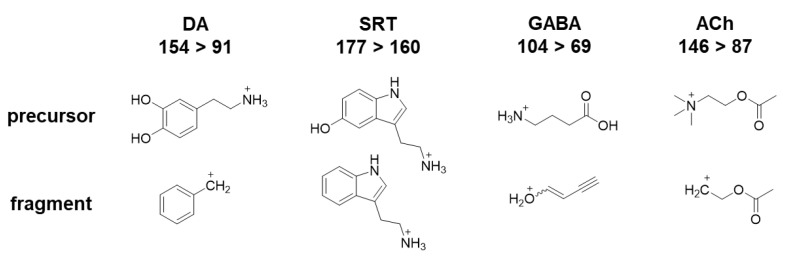
Chemical structures of precursors and their underlying fragment ions [M + H]^+^ (quantifiers) for DA, SRT, GABA and ACh.

Ion source parameters were optimized with standard solutions using the Compound Optimization software wizard of the Sciex Analyst Software (Version 1.7.2); they are listed in the materials and methods Section 2.4. To determine mass-to-charge (*m*/*z*) ratios for the precursor ions, standard solutions of the analytes and deuterated analytes were injected and Q1 scans were performed. Fragment ion scans with varying intensity in collision energy were conducted to determine the *m*/*z* ratios of the respective fragments. The aim was to identify at least two MRM transitions for each analyte with optimal intensity. The following mass transitions revealed the highest responses (Figure 1C–F) and were therefore used as quantifiers: DA *m*/*z* 154 > 91, DA_d4_ *m*/*z* 158 > 95, SRT *m*/*z* 177 > 160, SRT_d4_ *m*/*z* 181 > 164, GABA *m*/*z* 104 > 69, GABA_d6_ *m*/*z* 110 > 73, ACh *m*/*z* 146 > 87 and ACh_d4_ *m*/*z* 150 > 91. Quantifier precursor and fragment ion structures are stated in Figure 2. Further mass transitions (qualifiers) are listed in Table 1.

### 2.2. Sample Preparation and Neurotransmitter Extraction

Following optimization of the LC-MS/MS conditions, the method was applied to *C. elegans* homogenates. The extraction of neurotransmitters was improved by optimizing the composition of the applied extraction buffer. We tested the pH effect (acidic and neutral) on the stability and recovery of all four analytes. Whereas DA seems to be stable only in acidic pH, GABA shows the highest recovery in neutral pH. In contrast, both SRT and ACh demonstrate no differences in recovery in acidic or neutral pH. In order to identify a suitable compromise, various acids (perchloric acid and formic acid) and pH values (pH = 1–7) were tested. A sufficient response of all four analytes was obtained by adding 2.5 mM perchloric acid (pH = 4). In addition, we analyzed the impact of different amounts (10, 20 and 30%) of organic modifiers (MeOH and ACN) in the extraction buffer. A higher response, especially for GABA, was observed when we modified the buffer with 10% MeOH. The sample extracts were purified by a Spin-X^®^ Centrifuge Tube Filter 0.22 µm (Corning). The recovery of the neurotransmitters as well as the protein content with and without purification steps were determined, and showed statistically indistinguishable results.

### 2.3. Method Validation

**Figure 3:**
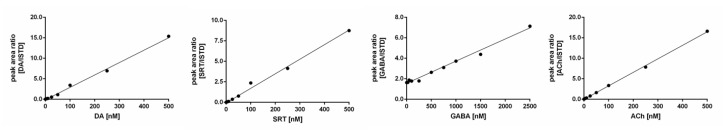
Calibration curves for all four neurotransmitters in the concentration range of up to 500 nM for DA, SRT and ACh and up to 2500 nM for GABA. Correlation coefficients are stated in Table 2.

Samples were spiked with DA, SRT and ACh from 0 to 500 nM and with GABA from 0 to 10 µM. Linearity was observed for all analytes in the indicated range (Figure 3); correlation coefficients are listed in Table 2.

The LOD was defined as LOD = 3×SD_y_/b (SD_y_ = standard deviation of analyte concentration in ≥12 blank measurements, b = slope of calibration curve), with 0.204 nM for DA, 0.097 nM for SRT, 15.628 nM for GABA and 0.0009 nM for ACh. The LOQ was defined as LOQ = 10×SD_y_/b, with 0.679 nM for DA, 0.324 nM for SRT, 52.094 nM for GABA and 0.0029 nM for ACh. Thus, the LOQs were far below the analyte concentrations in *C. elegans* homogenates (3000 L4 stage worms in 150 µL extraction buffer), which were 11.9 nM of DA, 2.2 nM SRT, 2.6 µM GABA and 38.8 nM ACh (n ≥ 20). The LOQs underline the sensitivity of the method and show that considerably less than 3000 worms can be used for the analysis. The recovery of deuterated standards in matrix amounted to 103 ± 3% for DA, 64 ± 2% SRT, 80 ± 4% for GABA and 56 ± 12% for ACh, compared to deuterated standards in extraction buffer only. This indicates sufficient recovery, as the loss of neurotransmitters during sample preparation and analysis was always balanced by the respective deuterated standards.

Accuracy was determined in samples with low (25 nM), middle (250 nM) and high (2500 nM) concentrations of all analytes and was within ± 20% of the nominal concentration (Table 3). The variation in neurotransmitter quantification from eight samples on the same day was defined as intraday precision and was 3.1% for DA, 6.1% for SRT, 3.4% for GABA and 7.6% for ACh. The variation from eight samples analyzed on different days was defined as interday precision and was 2.6% for DA, 14.0% for SRT, 3.2% for GABA and 1.8% for ACh. Therefore, intra- and interday variations < 15% were considered both reliable and reproducible due to high precision.

### 2.4. Neurotransmitter Levels in Wildtype Worms and cat-2Δ and ace-1Δ::ace-2Δ Deletion Mutants

By using the validated LC-MS/MS method, we investigated the impact of the genetic background of *C. elegans* strains cat-2Δ and ace-1Δ::ace-2Δ on neurotransmitter levels. The deletion mutant cat-2Δ lacks the enzyme tyrosine hydroxylase, which catalyzes the hydroxylation of tyrosine to l-DOPA (l-3,4-dihydroxyphenylalanine), the precursor of DA [43]. Consequently, DA synthesis in cat-2Δ worms is restricted. *C. elegans* strain ace-1Δ::ace-2Δ displays a loss of acetylcholinesterase (AChE), which is the major enzyme to hydrolyze ACh into acetic acid and choline [44]. As a result, this deletion mutant should not be capable of degrading ACh.

**Figure 4:**
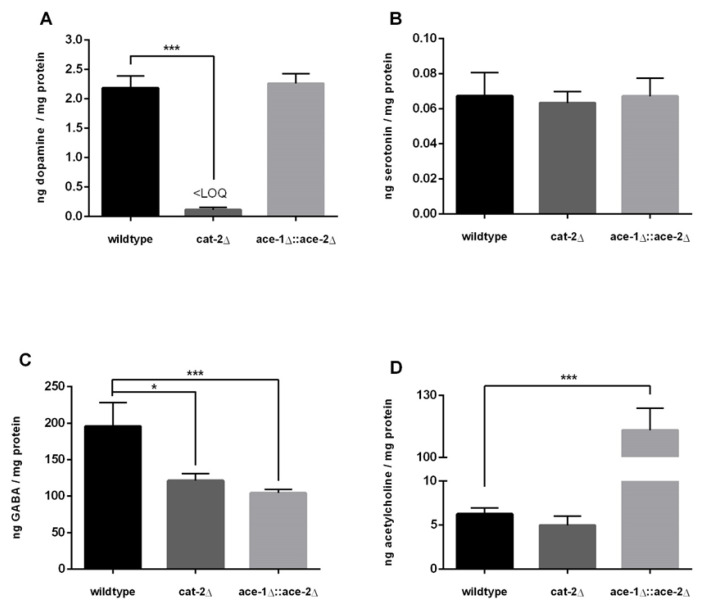
Levels in ng per mg protein of dopamine (**A**), serotonin (**B**), GABA (**C**) and acetylcholine (**D**) in L4 stage worms (wildtype, cat-2Δ and ace-1Δ::ace-2Δ) quantified via LC-MS/MS. Data presented are mean values of n = 4 independent experiments + SEM. Statistical analysis using unpaired *t*-test. Significance levels with α = 0.05: *: *p* ≤ 0.05 and ***: *p* ≤ 0.001 compared to wildtype worms.

The analysis of dopamine levels (Figure 4A) revealed 2.18 ± 0.19 ng DA per mg protein in wildtype worms and 2.26 ± 0.15 ng DA per mg protein in ace-1Δ::ace-2Δ deletion mutants. cat-2Δ worms displayed 0.11 ± 0.04 ng DA per mg protein or 0.54 nM DA in sample extracts; thus, DA levels were significantly lower compared to wildtype worms. This demonstrates that cat-2Δ worms do not suffer a total loss of DA, but nevertheless present a very low level of DA, which is higher than the LOD, but lower than the LOQ. As a result, cat-2Δ worms exhibited 95% less DA compared to wildtype worms. The quantification of SRT revealed no differences in the deletion mutants used compared to wildtype worms. SRT levels (Figure 4B) amounted to 0.067 ± 0.012 ng SRT per mg protein in wildtype worms, 0.063 ± 0.006 ng SRT per mg protein in cat-2Δ worms and 0.067 ± 0.009 ng SRT per mg protein in ace-1Δ::ace-2Δ worms. Wildtype worms contained 196 ± 30 ng GABA per mg protein (Figure 4C). Interestingly, cat-2Δ worms displayed a significantly lower amount of 121 ± 9 ng GABA per mg protein, whereas the deletion mutant ace-1Δ::ace-2Δ had the lowest amount of 104 ± 4 ng GABA per mg protein, which significantly differed compared to wildtype worms. The next neurotransmitter we quantified was ACh (Figure 4D); 6.24 ± 0.64 ng ACh was contained per mg protein in wildtype worms and 4.97 ng ACh per mg protein in cat-2Δ worms, representing a slight decrease, although it was statistically indistinguishable from wildtype worms. In contrast, the deletion mutant ace-1Δ::ace-2Δ contained a significantly higher amount of ACh compared to wildtype worms, with 113 ± 9 ng ACh per mg protein. Thus, ace-1Δ::ace-2Δ worms contained 18-fold greater ACh levels compared to wildtype worms.

### 2.5. Aldicarb-Induced Paralysis Assay

To investigate the consequences of our findings regarding the neurotransmitter quantification of the two deletion mutants, cat-2Δ and ace-1Δ::ace-2Δ, compared to wildtype worms, a classical applied behavioral assay was performed. Aldicarb is an AChE inhibitor, which leads to an accumulation of ACh, and therefore to a persistent activation of muscles followed by paralysis. The aldicarb-induced paralysis assay examines alterations in the synaptic transmission of *C. elegans* [45]. Aldicarb resistance, compared to wildtype worms, results in decreased synaptic transmission. By implication, aldicarb hypersensitivity leads to increased synaptic transmission [46].

**Figure 5:**
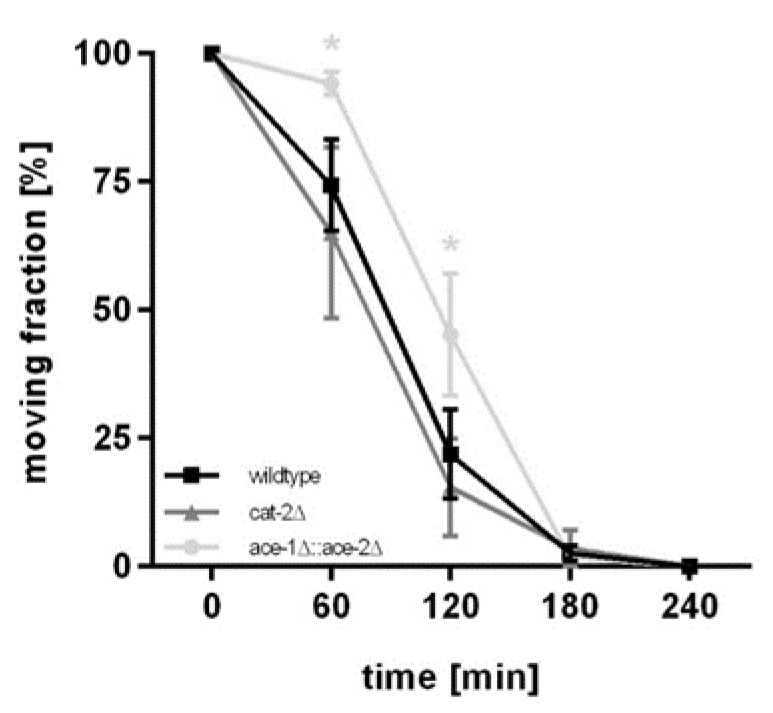
Aldicarb-induced paralysis assays in wildtype worms (black), cat-2Δ (dark grey) and ace-1Δ::ace-2Δ (light grey) deletion mutants. Displayed are fractions of moving worms [%] plotted against assay procedure times [min]. Data presented are mean values of n = 4 independent experiments ± SEM. Statistical analysis using unpaired *t*-test. Significance levels with α = 0.05: *: *p* ≤ 0.05 compared to wildtype worms at the same time point.

Results are presented in Figure 5 and demonstrate the paralysis rate in all three tested worm strains over a time span of 240 min. The cat-2Δ strain showed an earlier onset of paralysis compared to wildtype worms, with only 65% ± 14% of worms moving after 60 min (wildtype: 74% ± 7%) and 15% ± 8% after 120 min (wildtype: 22% ± 7%) when exposed to aldicarb, but the difference did not attain statistical significance. ace-1Δ::ace-2Δ worms, in contrast, showed significant aldicarb resistance compared to wildtype worms, with 94% ± 2% of worms moving after 60 min and 45% ± 10% after 120 min of aldicarb exposure. Taken together, these findings establish that the loss of AChE leads to reduced synaptic transmission in *C. elegans* due to aldicarb resistance.

## 3. Discussion

Tight regulation of the neurotransmitters is required to avoid adverse consequences of deficiency or excess, since various neurological diseases are characterized by a disturbed neurotransmitter homeostasis. Diseases associated with dysregulated neurotransmitters include PD, AD or depression, among others [47]. In this context, it is important to underline that in most clinical disorders, more than a single neurotransmitter is altered in its homeostasis [48,49]. Therefore, we have developed an LC-MS/MS-based method to simultaneously quantify multiple neurotransmitters within a single sample and run, which allows the quantification of DA, SRT, GABA and ACh, as well as the identification of potential changes in neurotransmitter ratios. It is important to note that this, to our knowledge, is the first method proposed to quantify multiple neurotransmitters, especially GABA and ACh, in *C. elegans*. To verify the optimized and validated method, we took advantage of the fact that *C. elegans* is easily genetically manipulated, and used worms that cannot synthesize DA (cat-2Δ) or degrade ACh (ace-1Δ::ace-2Δ), analyzed their neurotransmitter profiles, and characterized their impacts on synaptic transmission by a further independent assay, which refers to classically performed behavioral assays.

Our method for neurotransmitter quantification distinguishes itself from other published MS-based methods given its advantages. First, only a low quantity of worms is necessary for an analysis. Furthermore, there is only minimal sample preparation required, as the extraction buffer has been optimized regarding pH and organic modifiers for all analytes, so further time-consuming extraction steps are not required. The pH value of the buffer used is particularly important for neurotransmitter extraction, since DA autoxidizes easily at a neutral pH value [50], and extraction must therefore take place in an acidified milieu. GABA, on the other hand, displayed the best extraction in the neutral to slightly acidic pH range, with the result that we found a good compromise of pH = 4 for maximal extraction, which provides a higher overall sensitivity. The limits of detection for all analytes in matrix are in the very low nM range, which is advantageous compared to other LC-MS-based methods for the quantification of neurotransmitters in other matrices, as well as in standard solutions only [39,41,51,52]. Tufi et al. present an LOQ for SRT of 1.7 nM in zebrafish *Danio rerio*, which is roughly comparable to our data, whereas for other neurotransmitters like DA and GABA, two-digit nM quantification limits are displayed [41]. Huang et al. and Wang et al.’s LOQ for GABA in mice brain tissue is lower than that presented by us at 10 nM; however, their LOQs for DA and SRT are above 1 nM, and thus higher than those demonstrated in our study [15,51]. Olesti et al. demonstrate LOQs in the two-digit nM range in rat plasma and brain homogenates [52], while in Blanco et al.’s study, the average values of DA and ACh in mouse cerebrospinal fluid are below the LOQ [16]. However, the sensitivity is increased enough in the presently evaluated method to quantify the four neurotransmitters in a few 100 worms, which would allow high-throughput analyses in order to identify, for example, neurotoxic or neuroprotective substances. The method also offers high accuracy, as we use the respective isotope-labeled standards for each analyte throughout the entire sample preparation to compensate for losses in recovery and allow for an unequivocal identification of the neurotransmitters. The use of isotope-labeled standards is also a special feature of this method, which is often unconsidered [15,40,53]. Another advantage that underlines the specificity of our method is the use of a tandem mass spectrometer. The fragment pattern, characterized by the *m*/*z* ratios of the precursor ion and fragment ions, is as unique as a fingerprint for each molecule [54] and enables us to specifically identify our targeted analytes, rather than using retention times only. Other types of detection, such as quantification by fluorescent dyes [35,36,37], are less specific than the method described herein. Neurotransmitter quantification by HPLC with fluorescence detection is also both less specific and less sensitive, since it is necessary to derivatize the analytes into a fluorescent product. In addition, external calibration is commonly necessary [33,34]. In addition, other methods, such as that proposed by Zhang et al., combine precolumn derivatization with LC-MS/MS analysis to increase the specificity and sensitivity [55]. This provides LOQs in the single-digit nM range comparable to those produced by our method, but an additional derivatization step must be performed, which bears a further opportunity for error and takes another 30 min.

The roundworm *C. elegans* has become a prominent model organism and multipurpose tool to study neurotoxicity. Since only very few neurodegenerative diseases are linked to genetic factors, growing evidence strongly implicates environmental factors in their respective etiology. Therefore, the worm, with its existing neurodegenerative disease models (mostly transgenic worms), offers the opportunity for testing potential neurodegenerative substances and treatments, which may reflect or even accelerate the progression of neurodegenerative disorders. The quantification of neurotransmitter levels allows for precise identification of mechanisms that mediate neurotoxicity, and identifies putative targets for efficient therapeutic approaches and neuroprotective strategies. A special feature of *C. elegans* is its short life cycle, which allows a huge sample quantity in a short time period, and in combination with the presented analysis offers an effective high-throughput method. A further advantage of the worm is its completely sequenced genome, allowing its simple genetic manipulation. As a result, especially for neurobehavioral assays, chemicals or toxins are often not used as positive controls; rather, worms with specific mutations are. A commonly used assay is the basal slowing response, which examines dopamine-dependent behavior in the presence of food [25]. cat-2Δ worms are a popular positive control, since they show reduced food sensing due to its deficiency in DA synthesis [56,57]. Mutations of *C. elegans* are also often used to model neurodegenerative diseases like PD [21] and AD [23]. Despite the extensive use of mutants of this worm in neurobehavioral assays, its neurotransmitter profile has not been characterized, to our knowledge. Despite the usage of behavioral assays and the microscopy of fluorescence-tagged neurons, only a few chromatographic approaches have been carried out in *C. elegans* to quantify DA. Only Schumacher et al. displayed a validated LC-MS/MS-based method to analyze DA and SRT in *C. elegans*, but they excluded GABA and ACh [30], which are, however, essential for the investigation of neurotoxicity [10,58]. Using our method, we were able to determine neurotransmitter profiles in wildtype worms, as well as in cat-2Δ and ace-1Δ::ace-2Δ worms. As suggested in the literature, cat-2Δ worms had lesser DA levels compared to wildtype worms, which was corroborated by our LC-MS/MS method. In addition, we could also identify altered GABA levels. The same applied to ace-1Δ::ace-2Δ worms, wherein we could detect increased ACh levels as expected, but also reduced GABA levels, which underlines the interdependence and homeostatic dependence of different neurotransmitters. Muñoz et al. demonstrated interactions between the dopaminergic and serotonergic systems in PD [59]. Qi et al. reported how different neurotransmitters modulate neurotransmitter balance, and therefore regulate the function of different brain regions [13]. This emphasizes the importance of simultaneously quantifying multiple neurotransmitters, which has been achieved with this LC-MS/MS-based method. In contrast, behavioral assays do not constitute quantitative methods, but merely provide an insight into the consequences of an eventual neurotransmitter dyshomeostasis. It is noteworthy that the combination of instrumental analytics (especially mass spectrometry) and behavioral assays complement each other remarkably well. Therefore, the worm strains mentioned above were subjected to the aldicarb-induced paralysis assay in addition to neurotransmitter quantification.

Aldicarb, an AChE inhibitor, promotes the accumulation of ACh in locomotor neuromuscular junctions in *C. elegans* [60]. This results in hyperexcitability and excessive muscle contraction, causing paralysis [61]. If a mutant strain displays higher ACh levels, it should undergo paralysis faster. However, it has been shown that not only ACh itself is involved in aldicarb-induced paralysis, but the entire cholinergic system. Upon aldicarb treatment, mutants with impaired cholinergic function accumulate synaptic ACh at a slower rate, resulting in slower paralysis, and therefore aldicarb resistance, compared to wildtype worms [62].

This is consistent with our data, where ace-1Δ::ace-2Δ mutants showed a slower onset of paralysis, which was also demonstrated by Oppermann and Chang [63]. Hypothetically, it is not an increase in total ACh levels that leads to the onset of paralysis, but increased ACh levels in the neuromuscular junction. Giles et al. [62] reported that worms with disrupted inhibitory GABA function had a faster paralysis rate due to a loss of relaxation. Thus, given the GABA deficiency, cat-2Δ worms should paralyze faster in the presence of aldicarb compared to wildtype worms, which does not appear to be the case. It appears that behavior is a not fully understood yet complex construct in *C. elegans*, and further research is required to understand the underlying mechanisms of behavioral assays like the aldicarb-induced paralysis assay. This underscores that the combination of behavioral assays for *C. elegans* and the quantitative and validated methods such as the LC-MS/MS-based method developed herein provide the means for altered functional characterization along with its underpinning mechanisms. It is also noteworthy that the behavioral assays mentioned are species-specific, in this case *C. elegans*-specific. However, our LC-MS/MS method for the quantification of neurotransmitters is universally applicable and can be applied to other model systems and tissues in the future with the eventual adaption of sample preparation.

## 4. Materials and Methods

### 4.1. C. elegans Handling and Cultivation

*C. elegans* strains Bristol N2 (wildtype) and deletion mutants (Δ) CB1112 (cat-2Δ) and GG201 (ace-1Δ::ace-2Δ) were obtained from the Caenorhabditis Genetics Center (CGC, Minneapolis, MN, USA), which is funded by the National Institutes of Health Office of Research Infrastructure Programs. Cultivation of *C. elegans* was maintained on 8P agar plates coated with the *Escherichia coli* (*E. coli*) strain NA22 at 20 °C as previously described [64,65]. To generate age-synchronous worm populations, gravid adults were treated with bleach solution (1% NaOCl and 0.5 M NaOH) to release eggs, which were allowed to hatch overnight in M9 buffer. Synchronous L1-stage larvae were placed on nematode growth (NGM) agar plates coated with *E. coli* strain OP50 for 48 h to reach L4 stage.

### 4.2. Neurotransmitter Standard Solutions

Dopamine hydrochloride (Alfa Aesar, Kandel, Germany) and 2-(3,4-dihydroxy-phenyl)ethyl-1,1,2,2-d4-amine HCl (DA_d4_) (CDN Isotopes, Pointe-Claire, Canada) were dissolved in 200 mM HClO_4_ (Sigma-Aldrich, Steinheim, Germany), whereas γ-aminobutyric acid (Sigma-Aldrich, Steinheim, Germany) and 4-aminobutyric-2,2,3,3,4,4-d6 acid (GABA_d6_) (EQ Laboratories GmbH, Augsburg, Germany) stock solutions were prepared in 10% methanol (MeOH) (LC-MS grade, Thermo Fisher Scientific, Waltham, MA, USA). Serotonin hydrochloride (Alfa Aesar), serotonin-α,α,β,β-d4 creatinine sulfate complex (SRT_d4_) (CDN Isotopes, Pointe-Claire, Canada), acetylcholine chloride (Sigma-Aldrich, Steinheim, Germany) and acetylcholine-1,1,2,2-d4 chloride (ACh_d4_) (EQ Laboratories GmbH, Augsburg, Germany) were dissolved in bidistilled water. The deuterated analogue of the respective neurotransmitter was taken as an internal standard.

### 4.3. Sample Preparation and Neurotransmitter Extraction

Synchronous L4 stage wildtype, cat-2Δ and ace-1Δ::ace-2Δ worms were washed off from NGM agar plates using 85 mM NaCl + 0.01% Tween. The washing procedure was repeated three times to ensure samples were free of *E. coli*. Of each respective strain, 3000 worms were pelletized in 50 µL 85 mM NaCl by centrifugation at 380 g, frozen in liquid nitrogen and stored at −80 °C. Extraction buffer (2 mM sodium thiosulfate, 2.5 mM HClO_4_, 10% MeOH LC-MS grade, 25 mM DA_d4_, 25 mM SRT_d4_, 25 mM ACh_d4_ and 500 mM GABA_d6_) was freshly prepared right before sample preparation. Samples were kept on ice during sample preparation and extracted samples were analyzed immediately by LC-MS/MS. In the first step, worm pellets were defrosted and 100 µL extraction buffer was added, as well as zirconia beads (biolab products, Bebensee, Germany). To homogenize the samples: 4× freeze–thaw cycles (1 min 37 °C, 1 min liquid nitrogen) followed by 4 × 20 sec bead beating by usage of a Bead Ruptor (biolab products, Bebensee, Germany). After centrifugation for 10 min at 16,060× *g* at 4 °C, 100 µL of the supernatant was transferred to a Spin-X^®^ Centrifuge Tube Filter 0.22 µm (Corning, Amsterdam, The Netherlands) and centrifugation was repeated. An aliquot was transferred to a vial with insert and analyzed via LC-MS/MS, while the rest was used for protein quantification for normalization measured by bicinchoninic acid assay [66].

### 4.4. LC-MS/MS Parameters

All analyses were conducted using an Agilent 1290 Infinity II liquid chromatography system (Agilent, Waldbronn, Germany) coupled with a Sciex QTRAP 6500+ triple quadrupole mass spectrometer (Sciex, Darmstadt, Germany) interfaced with an electrospray ion source, which operated in positive ion mode. Chromatographic separation was performed using a YMC-Triart PFP (pentafluorophenyl) column (3 µm, 3 × 150 mm) and an additional precolumn (3 µm, 3 × 10 mm) of the same column material. The elution of neurotransmitters was carried out with bidistilled water + 10 mM formic acid (FA) (LC-MS grade, Thermo Fisher Scientific, Waltham, MA, USA) and acetonitrile (ACN) (LC-MS grade, VWR, Darmstadt, Germany) + 10 mM FA. Three µL of the sample was injected. Analytes were eluted with a flow of 0.425 mL min^−1^ from the column, which was pre-heated to 30 °C. Total run time was 12 min, which was divided in a gradient with 0% ACN for 3 min, 0 to 60% ACN for 6 min, 60 to 100% ACN for 0.5 min, 100% ACN for another 0.5 min, 100 to 0% ACN for 0.5 min and 0% ACN for re-equilibration for 1.5 min. Analysis was carried out in scheduled multiple reaction monitoring (sMRM) mode with detection windows of ±40 sec of the respective retention times (Table 1). Ion source parameters optimization was performed with standard solutions of DA, SRT, GABA and ACh using the *Compound Optimization software wizard* of the Sciex Analyst Software (Version 1.7.2). The following parameters were determined: ion spray voltage = 4000 V, curtain gas (N2) = 40 psi, nebulizer gas = 60 psi, drying gas = 50 psi, collision (CAD) gas = medium, temperature = 600 °C, entrance potential = 10 V. The dwell time for all analytes and deuterated standards was set to 20 ms. Mass transitions for the analytes and internal standards as well as the respective optimized collision energy (CE), declustering potential (DP) and collision cell exit potential (CXP) are listed in Table 1.

### 4.5. Method Validation

Method validation was carried out according the “ICH guideline Q2(R2) on validation of analytical procedures” of the European Medicines Agency. Linear range, limit of detection (LOD), limit of quantification (LOQ), recovery, accuracy and intraday and interday precision were assessed for method validation. To investigate linear ranges, LODs and LOQs, solutions with fixed amounts of matrix (wildtype) and deuterated internal standards were added with neurotransmitter standards in a range of 0–500 nM for DA, SRT and ACh and 0–10 µM for GABA, and analyzed twice. Peak areas of the analytes were normalized to the respective internal standards, plotted against the added concentrations and afterwards examined for linear correlation. Signal-to-noise ratios (S/N) were calculated using *Multiquant Software* (Sciex, Version 3.0.3) and plotted against the added concentrations; the slopes were determined subsequently. LOD and LOQ were defined as LOD = 3 × SD_y_/b (SD_y_ = standard deviation of analyte concentration in ≥12 blank measurements, b = slope of calibration curve) and LOQ = 10 × SD_y_/b. To assess the recovery of the deuterated internal standards in matrix, eight samples containing extraction buffer only and eight samples with worm matrix in extraction buffer were analyzed. Recovery was defined as the ratio of the area of internal standards with to that without matrix. For accuracy, matrix-free samples with low (25 nM), middle (250 nM) and high (2.5 µM) amounts of all neurotransmitters added along with 250 nM of deuterated standards were analyzed twelve times. Accuracy was calculated in percent by how much of the neurotransmitters was actually detected. To determine precision, intraday variation of eight wildtype worm samples pelletized and analyzed on the same day, and interday variation of six wildtype worm samples, each pelletized and analyzed on six different days, were assessed. Samples were normalized for protein content for examination of intraday as well as interday precision. Precision is stated as relative standard deviation in percent (RSD%) of the above-mentioned samples.

### 4.6. Aldicarb-Induced Paralysis Assay

Synchronous L1 stage worms were placed on NGM plates as mentioned above for 72 h until the young adult stage. The assay was performed based on Mahoney et al. [27]. In brief, a 100 mM aldicarb (Sigma-Aldrich, Steinheim, Germany) stock solution was prepared in 70% ethanol. For plates with 2 mM aldicarb, NGM agar was set up as previously described [42,43] and added with aldicarb for desired concentration. Three mL portions were poured into 3.5 cm petri dishes and stored at 4 °C. The plates were coated at the very beginning of the experiment with 2 µL of *E. coli* strain OP50 to concentrate worms in the middle of the plates. The assay was always performed as a blinded experiment. Of each genotype, 20–25 worms were placed on an aldicarb-containing plate, which were left at room temperature during the assay procedure. Every 60 min, the number of total and paralyzed worms was counted. Worms were defined as paralyzed if they demonstrated no movement after prodding carefully with a platinum wire against head and tail.

### 4.7. Statistics

Statistical analysis was performed using GraphPad Prism 6 (GraphPad Software, La Jolla, CA, USA) via unpaired *t*-test. Significance levels with α = 0.05 are depicted as *: *p* ≤ 0.05, **: *p* ≤ 0.01 and ***: *p* ≤ 0.001, compared to wildtype worms.

## 5. Conclusions

In summary, here (1) we developed a novel liquid chromatography–tandem mass spectrometry (LC-MS/MS) method, which enables simultaneous neurotransmitter quantification of dopamine (DA), serotonin (SRT), γ-aminobutyric acid (GABA) and acetylcholine (ACh) in the nematode, *C. elegans*, an assay (2) which can readily be applied to other matrices. (3) Furthermore, the LC-MS/MS method combined with isotope-labeled standards provides exquisite sensitivity and specificity, (4) providing a validated analytical method for the assessment of altered neurotransmission and neurotoxicity. Our analytical method allows the quantification of neurotransmitters and their ratios as a convenient tool for the identification of mechanisms that mediate neurotoxicity, and it should be helpful in identifying possible putative therapeutic approaches and targets. Neurotoxicity assessment in *C. elegans* is commonly carried out by behavioral assays, which provide a sensitive assay for altered neurological behaviors, but are unable to characterize neurotransmitter levels. Other than *C. elegans* species-specific behavioral assays, our method is equally applicable to other tissues and matrices.

## Figures and Tables

**Table 1:** sMRM parameters for DA, SRT, GABA, ACh and their respective internal standards. The quantifiers are highlighted in bold. All transitions are single-protonated ions ([M + H]^+^).

Compound	Q1	Q3	CE	DP	CXP	Retention Time (min)
DA	154	137	15	30	15	5.92
		119	25	30	15	
		**91**	32	30	15	
DA_d4_	158	141	15	30	15	
		123	25	30	15	
		**95**	32	30	15	
SRT	177	**160**	15	15	17	8.38
		115	51	30	41	
SRT_d4_	181	**164**	15	15	17	
		118	51	30	41	
GABA	104	87	15	17	10	2.50
		**69**	21	18	10	
		45	28	25	11	
GABA_d6_	110	93	15	17	10	
		**73**	21	18	10	
		49	28	25	11	
ACh	146	**87**	19	27	13	7.22
		60	16	32	9	
ACh_d4_	150	**91**	19	27	13	
		60	16	32	9	

**Table 2:** Method validation parameters assessed in *C. elegans* matrix (wildtype). How parameters were assessed is listed in Section 4.5.

	DA	SRT	GABA	ACh
Concentration in samples ^#^	11.9 nM	2.2 nM	2.6 µM	38.8 nM
Correlation coefficient (R^2^)	0.9966	0.9939	0.9873	0.9993
Limit of detection (nM)	0.204	0.097	15.628	0.0009
Limit of quantification (nM)	0.679	0.324	52.094	0.0029
Recovery (%)	103 ± 2.7	64 ± 2.3	80 ± 4.1	56 ± 11.9

^#^ analyte concentration of worm homogenates (3000 L4 stage worms in 150 µL extraction buffer) before protein normalization.

**Table 3:** Method validation parameters: accuracy for low, middle and high analyte concentrations and intraday and interday precision. How parameters were assessed is listed in Section 4.5.

	Accuracy [%]			Precision [RSD%]	
	Low	Middle	High	Intraday	Interday
DA	114.8 ± 8.8	111.1 ± 7.9	112.7 ± 4.1	3.1	2.6
SRT	84.9 ± 1.3	85.6 ± 1.5	81.1 ± 1.8	6.1	14.0
GABA	95.3 ± 8.7	108.2 ± 5.2	116.4 ± 5.4	3.4	3.2
ACh	98.5 ± 4.9	96.6 ± 1.0	99.8 ± 0.6	7.6	1.8

## Data Availability

The data that support the findings of this study are available from the corresponding author upon reasonable request.
